# Epidemiology of Asymptomatic Pre-heart Failure: a Systematic Review

**DOI:** 10.1007/s11897-022-00542-5

**Published:** 2022-03-30

**Authors:** Aurore Bergamasco, Anouk Luyet-Déruaz, Nicholas D. Gollop, Yola Moride, Qing Qiao

**Affiliations:** 1 YolaRX Consultants, Paris, France; 2 Boehringer Ingelheim International GmbH, Ingelheim Am Rhein, Germany; 3 YolaRX Consultants, Montreal, Canada; 4 Faculty of Pharmacy, Université de Montréal, Montreal, Canada; 5 Rutgers, The State University of New Jersey, New Brunswick, NJ USA

**Keywords:** Disease progression, Mortality, Prevalence, Stage B heart failure

## Abstract

**Purpose of Review:**

To quantify the prevalence of asymptomatic pre-heart failure (pre-HF), progression to more severe stages, and associated mortality.

**Recent Findings:**

A systematic review was conducted between 01 January 2010 and 12 March 2020 (PROSPERO: CRD42020176141). Data of interest included prevalence, disease progression, and mortality rates. In total, 1030 sources were identified, of which, 12 reported on pre-HF (using the ACC/AHA definition for stage B HF) and were eligible. Prevalence estimates of pre-HF ranged from 11 to 42.7% (10 sources) with higher estimates found in the elderly, in patients with hypertension, and in men. Three studies reported on disease progression with follow-up ranging from 13 months to 7 years. The incidence of symptomatic HF (HF/advanced HF) ranged from 0.63 to 9.8%, and all-cause mortality from 1.6 to 5.4%.

**Summary:**

Further research is required to investigate whether early detection and intervention can slow or stop the progression from asymptomatic to symptomatic HF.

**Supplementary Information:**

The online version contains supplementary material available at 10.1007/s11897-022-00542-5.

## Introduction

Heart failure (HF) is a major public health issue and a leading cause of global morbidity and mortality [1]. An estimated 64.34 million individuals currently live with HF worldwide, contributing to 9.91 million years lived with disability [2], with numbers expected to increase in the future. The worldwide economic burden associated with HF is estimated at US $346.17 billion a year [3].

**Fig. 1:**
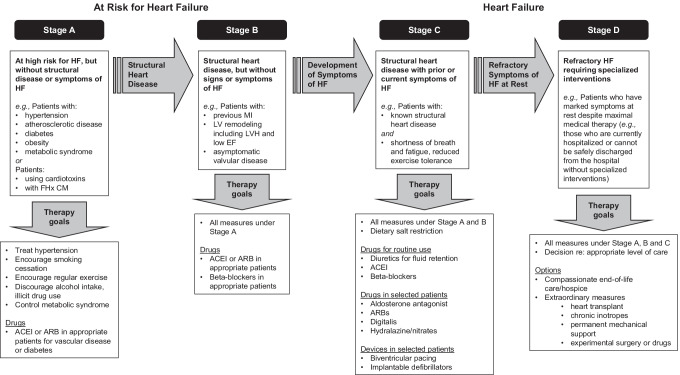
Evaluation and management of HF defined by ACC/AHA guidelines: evolution and recommended therapy by HF stage. Adapted from Hunt [50]. ACEI, angiotensin-converting enzyme inhibitors; ARB, angiotensin receptor blockers; EF, ejection fraction; FHx CM, family history of cardiomyopathy; HF, heart failure; LV, left ventricular LVH, left ventricular hypertrophy; MI, myocardial infarction

HF is a progressive disease that is classified by the American College of Cardiology (ACC) and American Heart Association (AHA) into four stages, based on the presence of structural remodeling and symptoms as shown in Fig. 1 [4•, 5•]. Briefly, patients are categorized as at risk of HF (stage A), with structural heart disease (stage B), with structural heart disease and symptoms of HF (stage C), or with refractory HF requiring specialized interventions (stage D) in which once a patient moves to a higher stage, they cannot revert to a lower stage [4•]. The ACC/AHA guidelines were developed to help identify patients with asymptomatic HF and to complement the clinically widely used NYHA classification which focuses solely on symptomatic HF. More recently, a universal definition of HF was published [6•], whereby they proposed the following stages: at risk for HF (ACC/AHA stage A), pre-HF (stage B), HF (stage C), and advanced HF (stage D). The proposed stages follow the same structure as the ACC/AHA staging system and address some of the gaps, namely the use of biomarkers to identify patients with structural disease, and clearly defined definitions for HF (e.g., abnormal cardiac function), which are now both included in the definition of pre-HF. The inclusion of stages A and B into a definition of HF may help with early detection and prevention of the progress from asymptomatic to symptomatic HF.

Asymptomatic HF includes patients at risk for HF and patients with pre-HF [4•]. Current estimates of the number of patients with asymptomatic HF vary widely but the number is thought to be higher than the number of patients with symptomatic HF (i.e., patients with HF or advanced HF) [7–9]. Patients with asymptomatic HF are at high risk of HF (including patients with hypertension, diabetes, and coronary artery disease) and can present with structural or functional cardiac abnormalities including previous myocardial infarction, left ventricle (LV) remodeling (including LV hypertrophy and low ejection fraction [EF]), and asymptomatic valvular disease [4•]. Other structural changes include asymptomatic LV systolic dysfunction (ALVSD) and asymptomatic LV diastolic dysfunction (ALVDD) [5•]. Studies tend to focus on ALVSD [10] rather than ALVDD which remains poorly understood [11].

Patients with structure cardiac dysfunction but without a clinical symptom are usually not detected unless an imaging assessment such as echocardiography is performed. There is increasing evidence for the use of natriuretic peptide (NP) biomarkers, such as B-type NP (BNP) and N-terminal pro-BNP (NT-proBNP), to detect the presence or severity of HF with a high diagnostic accuracy [12–16]. However, factors such as age, sex, race, renal dysfunction, and body mass index (among other cardiac/non-cardiac conditions) can affect the clearance of NPs [17–21], in which case, different cut-off levels are needed for different patient populations. Nevertheless, the use of BNP as a biomarker in the diagnosis of HF has been included in HF guidelines, including in the diagnosis of asymptomatic HF [22–26] and pre-HF [6•].

Asymptomatic HF in the general population is often unidentified unless a screening program is implemented with echocardiography; therefore, information on the epidemiology of asymptomatic HF in the general population is scarce. This systematic review aims to report the prevalence of asymptomatic in different patient groups and to describe its progression to symptomatic stages of HF and the associated rate of mortality. There are a number of definitions of asymptomatic HF and these definitions include a wide group of patients including those with no structural changes or symptoms. The ACC/AHA guidelines are widely used and provide clear guidance for identification of structural changes using echocardiogram providing an objective and uniform/standard definition for studying patients with asymptomatic HF. Therefore, we narrowed the focus of this review to concentrate on those studies reporting data on ACC/AHA stage B HF to minimize the bias in reported prevalence caused by differences in the definitions.

## Methods

A systematic review was conducted according to a study protocol developed a priori and registered in PROSPERO before the start of data extraction (CRD42020176141). The search period covered from 01 January 2010 to 12 March 2020.

### Search Strategy

To identify eligible studies, a literature search using MEDLINE and Embase accessed by Ovid was conducted. Pragmatic searches of the gray literature were conducted using Google and Google Scholar search engines. Relevant websites of learned or clinical societies and associated conferences were also screened, including HFSA, AHA, ESC, World Heart Federation (WHF), ACC Annual Scientific Sessions, ESC Annual Meeting, and the World Congress on Cardiology and Heart Diseases. Search strategies, presented in Supplementary Table 1 (Online Resource 1), combined the following concepts: disease of interest (HF/ventricular dysfunction/heart left ventricle failure AND asymptomatic diseases) and data of interest (incidence, prevalence, risk factors, disease progression, prognosis, hospitalization, mortality). Additionally, a manual search of references from all eligible studies and review articles was undertaken (i.e., snowballing).

### Study Selection

**Fig. 2:**
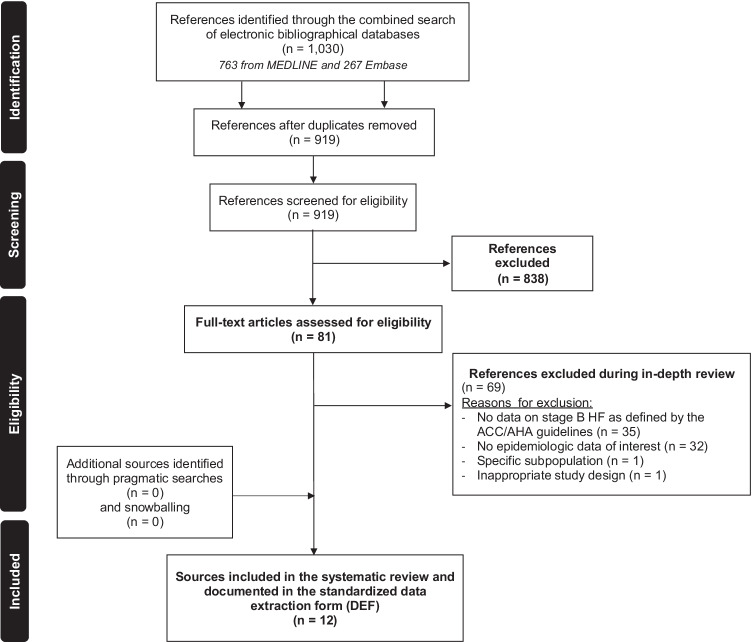
PRISMA flow chart of searches on the epidemiology of stage B HF, as defined by ACC/AHA guidelines

Observational (non-interventional) studies (including cohort, case–control, and cross-sectional studies) that included patients with pre-HF (stage B HF, as defined by the ACC/AHA guidelines) and that reported on data of interest (incidence, prevalence, risk factors, disease progression, prognosis, hospitalization, mortality) were retained. The definition of pre-HF includes patients with structural heart disease but no current or prior symptoms of HF such as patients with previous myocardial infarction, LV remodeling including LV hypertrophy and low EF, or asymptomatic valvular disease [27]. Original studies published as full-text or abstracts in English were considered and reviews were retained for snowballing only. The following publications were excluded: expert opinions, editorials, letters to editors, non-clinical and experimental studies, phase I–III clinical trials, case reports, case series, and literature reviews/meta-analyses (data was not extracted but used for snowballing only). Search outputs were screened, based on titles and abstracts, independently by two independent assessors with conflicts resolved by a third assessor. References retained after screening were reviewed in depth in order to confirm eligibility. Reasons for excluding studies at this stage were documented (e.g., no epidemiologic data of interest, no data on patients with pre-HF). A PRISMA flow chart illustrates the study selection process in Fig. 2.

### Data Extraction and Methodological Quality Assessment

Data extraction was performed independently by two reviewers using a standardized data extraction form that was piloted prior to the start of data extraction, using five relevant publications. Conflicts were resolved by a third senior assessor. For each retained study, information extracted included the following:

- General information
  - Mode of identification (literature search, pragmatic search, snowballing)
  - Study reference (first author and year of publication)
  - Type of source (abstract, conference proceeding, original study)
  - Geographic coverage (region, country[ies] covered)
- Study methods
  - Study period (or alternatively, year of publication if the study period is not reported)
  - Study design (prospective or retrospective cohort, case–control, cross-sectional study, etc.)
  - Data source (medical chart review, electronic medical records, administrative claims, registry, etc.)
  - Study population definition (inclusion/exclusion criteria)
  - Source population (general population, type 2 diabetes mellitus, other)
  - Case definition (diagnostic approach, diagnostic codes)
- Study results
  - Reference population (size and source of estimate — only for studies reporting incidence and/or prevalence estimates)
  - Sample size
  - Estimates of epidemiologic parameters (incidence of asymptomatic heart failure, prevalence of asymptomatic heart failure, frequency of undiagnosed heart failure, proportion of patients with controlled risk factors)
  - Burden of illness (hospitalization rate, cardiovascular complications, mortality [all-cause and cardiovascular-related])

The methodological quality of retained full-text publications was assessed using the Joanna Briggs Institute (JBI) critical appraisal tools for original studies [28], with the tools being specific to each type of study design. Studies were judged to be at low risk of bias (good quality) if they met at least 7 of 9 items of the checklist for cohort studies, 8 out of 10 items of the checklist for case series (appropriate for non-comparative cohort studies), or 8 out of 11 items of the checklist for studies reporting prevalence estimates. The review was conducted using the methods proposed by the Cochrane group [29] and the Institute of Medicine of the National Academy of Medicine [30].

## Results

### Study Selection and Study Characteristics

The selection of sources through the various phases of the search is summarized in a PRISMA flow chart (Fig. 2). A total of 1030 sources were identified through the literature search covered from 01 January 2010 to 12 March 2020. After screening, there were 81 full-text publications retained for in-depth review to confirm eligibility. During the in-depth review, 69 sources were further excluded, mainly because they did not report data on pre-HF (*n* = 35; 50.7%), or because they did not estimate the epidemiologic parameters of interest (*n* = 32; 46.4%). Pragmatic searches and snowballing yielded no additional sources. Data were thus extracted from 12 full-text articles.

From the 12 retained publications, there were 10 studies reporting prevalence [31••, 32••, 33••, 34••, 35••, 36••, 37••, 38••, 39••, 40••] with one of these also reporting disease progression and mortality [38••]. The two remaining studies were reporting disease progression and mortality only [41••, 42••]. Study designs consisted mainly of prospective cohort studies (*n* = 5; 41.7%), cross-sectional studies (*n* = 4; 33.3%), and retrospective cohort studies (*n* = 3; 25.0%). The majority of studies originated from Europe (*n* = 7; 58.3%) and North America (*n* = 3; 25.0%). Other sources covered Asia–Pacific (*n* = 1; 8.3%) and Latin America (*n* = 1; 8.3%). Study characteristics are summarized in Table 1, and details regarding definitions of pre-HF as reported in each publication are presented in Supplementary Table 2 (Online Resource 2).

**Table 1:** Characteristics of studies included in the systematic review on pre-HF (*N* = 12)

Study authors	Country	Year	Design	Study period	Data source	Source of estimates	Epidemiologic parameter
Europe (***n*** = 7)							
Smeets M et al. [31••]	Belgium	2019	RC	2000–2015	Registry (disease)	2000 Standardized Flemish population	Prevalence
Gaborit FS et al. [32••]	Denmark	2019	PC	Dec 2014–Jun 2016	Ad hoc data collection	Participants to the Copenhagen Heart Failure Risk Study	Prevalence
Ghossein-Doha C et al.[33••]	Netherlands	2017	CSS	2017*	Ad hoc data collection	Study participants	Prevalence
Breetveld NM et al.[34••]	Netherlands	2017	PC	2009–2011	Ad hoc data collection	Study participants	Prevalence
Pugliese N et al.[41••]	Italy	2018	RC	2018*	Medical chart review	Study participants	Disease progression Mortality
Mureddu GF et al.[35••]	Italy	2012	CSS	Jun 2007–Jan 2010	Ad hoc data collection	Participants to the PREDICTOR (Valutazione della PREvalenza di DIsfunzione Cardiaca asinTOmatica e di scompenso caRdiaco) Study	Prevalence
Mureddu GF et al.[36••]	Italy	2019	PC	Jun 2013–Oct 2013	Ad hoc data collection	Participants to the VASTISSIMO Study with available data to identify SBHF	Prevalence
North America (***n*** = 3)							
Shah AM et al.[37••]	US	2017	PC	2011–2013	Ad hoc data collection	Participants to the Atherosclerosis Risk in Communities (ARIC) Study	Prevalence
Xanthakis V et al.[38••]	US	2016	RC	2002–2008	Medical chart review	Participants to the Framingham Offspring and Framingham Third Generation cohort	Prevalence Disease progression Mortality
Gupta S et al.[39••]	US	2011	CSS	2011*	Ad hoc data collection	Participants to the Dallas Heart Study (DHS)	Prevalence
Asia–Pacific (***n*** = 1)							
Miura M et al.[42••]	Japan	2014	PC	Jan 2013–May 2013	Ad hoc data collection	Participants to the Chronic Heart Failure Analysis and Registry in the Tohoku District Study-2 (CHART-2)	Disease progression Mortality
Latin America (***n*** = 1)							
Jorge AL et al.[40••]	Brazil	2016	CSS	Jul 2011–Dec 2012	Ad hoc data collection	Study participants	Prevalence

*Abbreviations*: *CSS*, cross−sectional study; *PC*, prospective cohort; *RC*, retrospective cohort; *US*, United States. *Year of publication

Methodological quality was assessed for the ten full-text publications reporting prevalence estimates and is given in Supplementary Table 3 (Online Resource 3). Of these, 70% (*n* = 7) were considered to be of good methodological quality. Most studies (*n* = 8; 80%) that reported prevalence data used valid methods (i.e., echocardiography) to ascertain pre-HF thereby controlling for information bias. Also, 40% of studies (*n* = 4) used an expert panel to validate the diagnosis [32••, 35••, 37••, 38••].

### Prevalence of Pre-heart Failure

The key characteristics of the ten studies that reported estimates of the prevalence of pre-HF are summarized in Table 2. Of these, seven (70.0%) reported on prevalence in the general population [31••, 33••, 35••, 37••, 38••, 39••, 40••] and three (30%) included specific subpopulations such as patients with hypertension [32••], women with a history of pre-eclampsia [34••], or patients referred for an echocardiogram [36••].

**Table 2:** Prevalence estimates of pre-HF

Study reference	Source population	Sample size	Age (years)	Gender (male)	Diagnosis ascertained by an expert panel	Prevalence of pre-HF
Smeets M et al. 2019 [31••]	General population	165,796	≥ 45	Not reported	No	11%
Jorge AL et al. 2016 [40••]	General population	633	Mean ± SD: 59.6 ± 10.4	38%	No	42.7%
Mureddu GF et al. 2012 [35••]†	General population	2,001	65 to 84	51.7%	Yes	59.1%
Ghossein-Doha C et al. 2017 [33••]	General population	41	Mean ± SD: 40 ± 4	0%	No	7%
Shah AM et al. 2017 [37••]†	General population	6,118	Median (*IQR*): 75.3 (71.7–77.5)	42%	Yes	29.4%
Xanthakis V et al. 2016 [38••]†	General population	6,770	Mean ± SD: Men: 51 ± 16 Women:51 ± 16	46.0%	Yes	Overall: 24.2% Men: 30.9% Women: 18.5%
Gupta S et al. 2011 [39••]	General population	2,277	Mean ± SD: 47 ± 10	57%	No	12.5%
Gaborit FS et al. 2019 [32••]†	Patients with hypertension	400	Median (range): 72 (67–78)	51.5%	Yes	37.5%
Mureddu GF et al. 2019 [36••]	Patients referred for an echocardiogram	3,322	Mean ± SD: 67.1 ± 11.9	54.5%	No	52.6%
Breetveld NM et al. 2017 [34••]	Pre-eclamptic women	69	Median (*IQR*): 32 (29–35)	0%	No	23.2%

*Abbreviations*: *IQR*, interquartile range; *SD*, standard deviation; *US*, United States. †The investigators relied on an expert panel to validate the diagnosis of pre−HF

In the general population, the highest prevalence estimate was 59.1%, which originated from a cross-sectional study from Italy conducted in the elderly population (age 65–84 years) [35••]. The other prevalence estimates ranged from 11 [31••] to 42.7% [40••]. The lowest estimate of 11% corresponds to the age-standardized (to the Flemish population) prevalence of pre-HF, as reported in a retrospective cohort study conducted in Belgium (*n* = 165,796) [31••]. The higher estimate of 42.7% was found in a cross-sectional study conducted among 633 volunteers (mean age, 59.6 ± 10.4 years) identified from a primary care setting in Brazil [40••]. In a US population-based cross-sectional study of 2277 patients aged 30 to 65 years, the prevalence of pre-HF was estimated at 12.5% [39••]. An estimate of 24.2% was reported in a study based on data of the Framingham Offspring and Framingham Third Generation Cohort (*n* = 6770), which included older patients (mean age, 51 ± 16 years) over the period 2002–2008 [38••]. In addition, the prevalence of pre-HF among elderly patients (≥ 65 years) included in the Atherosclerosis Risk in Communities (ARIC) Study (*n* = 6118; median age, 75.4 years) was 29.2% [37••].

According to Table 2, the prevalence of pre-HF in the studies conducted in the general population increased with age. This is particularly apparent when comparing the studies conducted in the elderly population (prevalence, 59.1%; age, 56 to 84 years) [35••], or in a primary care setting (prevalence, 42.7%; mean age, 59.6 years) [40••] with studies conducted in the general Flemish population (prevalence, 11%; age, ≥ 45 years) [31••] or the general US population (prevalence 12.5%; mean age, 47 years) [39••]. Prevalence is increased in patients with hypertension [32••] or in women with pre-eclampsia [34••] when compared against studies conducted in the general population with a similar mean/median age. The prevalence in patients referred for an echocardiogram [36••] is also increased relative to studies conducted in the general population with a similar mean/median age, as patients are more likely to be diagnosed with pre-HF when they are monitored more closely. The prevalence of pre-HF was also higher in men than in women: 13% versus 10% in the Belgium study [32••] and 30.9% versus 18.5% in the US study [38••]. These findings were supported by results of a cross-sectional study of healthy women with previously uneventful pregnancy (mean age, 40 ± 4 years), which reported a prevalence of 7% [33••], although this study had a very small sample size (*n* = 41).

### Disease Progression in Patients with Pre-heart Failure

Data on the progression of HF from pre-HF to symptomatic HF (HF or advanced HF) in the literature is lacking. According to a large retrospective cohort study conducted in the USA, the incidence rate of new-onset symptomatic HF among patients with pre-HF (*n* = 1637) was estimated at 1.14 per 100 person-years in men and 0.63 per 100 person-years in women [38••]. In an Italian study that included patients with both at risk of HF or pre-HF (*n* = 337), 9.8% patients required hospitalization due to a transition from asymptomatic to new-onset (symptomatic) HF over a median follow-up of 22 months (interquartile range: 30–47 months) [41••]. In a prospective cohort study conducted in Japan, 1.1% of patients with pre-HF were admitted to the hospital for HF (HF or advanced HF) after a median follow-up of 12.7 months (Table 3) [42••].

**Table 3:** Disease progression and mortality of pre-HF patients

Study reference	Source population	Sample size	Age (years)	Gender (male)	Follow-up period	Disease progression	Mortality
Miura M et al. 2014 [42••]	Stage B HF patients	2,380	Not reported	Not reported	Median: 12.7 months	Hospital admission for HF (symptomatic): 1.1%	All-cause death: 1.6%
Pugliese N et al. 2018 [41••]	Stage A or B HF patients	337	Mean ± SD: 54.7 ± 13.7	53.1%	Median (*IQR*): 22 (30–47) months	Hospitalization for HF (symptomatic): 9.8%	Cardiac death: 0.6% Noncardiac death: 0.9%
Xanthakis V et al. 2016 [38••]	Stage B HF patients	1,637	Not reported	Not reported	Mean: 7 years	New-onset stage C/D HF: Men: 1.14 per 100 person-year Women: 0.63 per 100 person-year	All-cause death: 5.4% Crude annual mortality rate: 0.881 per 100 person-years

### Mortality in Patients with Pre-heart Failure

Only three studies reported mortality rates in patients with pre-HF. In the US general population (all age groups included), the all-cause mortality rate of patients with pre-HF was estimated at 0.881 per 100 person-years [38••]. In Japan, 1.6% of patients with pre-HF (*n* = 2380) died over a median follow-up of 12.7 months [42••]. In Italy, it was reported that 0.6% patients at risk or HF or pre-HF (*n* = 337) died of cardiac death and 0.9% of noncardiac death over a median follow-up of 22 months (*IQR*: 30–47 months) [41••].

## Discussion

This systematic review of 12 studies provides a synthesis of the existing evidence on the prevalence of pre-HF. While it is noted that multiple asymptomatic heart failure definitions exist, this study focused on publications using the ACC/AHA stage B HF definition to minimize the bias caused by different definitions, and as the ACC/AHA definition is most widely used [6•], this study can be applied directly to this large and under-studied patient group [27]. When comparing the prevalence of pre-HF to the prevalence of symptomatic HF using the same guidelines and in similar populations, the prevalence of pre-HF was five to ten times higher than that of symptomatic HF. In the USA, prevalence estimates of pre-HF ranged from 12.5 to 24.2% [38••, 39••] while the prevalence of symptomatic HF has been estimated at 2.2% by the American Heart Association [43]. As the latter association estimates that 6.5 million American individuals currently live with symptomatic HF, the number of patients with pre-HF may reach 50 million individuals in the USA, therefore underlining the importance of this condition in terms of public health [44]. This extrapolation also clearly highlights the fact that pre-HF patients are under-represented in clinical research. The lack of identification, but high prevalence, also means that there is an increasing global disease burden of undiagnosed asymptomatic HF patients, many of whom will progress to symptomatic disease requiring urgent intervention [27].

Identifying patients with pre-HF is relatively difficult as patients are unlikely to seek medical attention in the absence of symptoms. Screening the whole population would be inadvisable as the diagnosis of pre-HF requires echocardiography examinations, which are associated with high costs and the need of skilled technicians. In this context, developing screening programs targeted to specific subpopulations who are at increased risk of pre-HF such as patients with identified risk factors (i.e., hypertension, diabetes mellitus) or elderly individuals (≥ 65 years), should be considered in clinical practice. Screening programs in high-risk groups could include echocardiograms, exercise stress tests, or blood tests to quantify markers such as BNP or NT-Pro-BNP [27, 45]. However, screening the general population would lead to the occurrence of false positive and false negative findings, which may cause “harm” (be it physical or psychological) to healthy individuals. Also, screening would invariably lead to a significant increase in new referrals to specialist services (for assessment and/or diagnosis) and this would create a major burden on many, already over-subscribed, healthcare systems. Despite these challenges, the need to diagnose heart failure sooner is unequivocally needed [46, 47].

Data on the mortality associated with pre-HF are scarce in the literature. However, it has been well documented that patients with symptomatic HF are at increased risk of premature death compared to healthy individuals. The 1-year mortality rate following diagnosis of symptomatic HF in the Framingham study ranged from 26 to 35% over the period 1950–1999 [48]. As HF is a progressive disease, the vast majority of patients with pre-HF patients will ultimately develop symptomatic HF. Thus, early identification of these patients and implementation of targeted interventions aiming to reduce the risk of progression from asymptomatic pre-HF to symptomatic HF may help prevent premature death attributed to symptomatic HF. Current guidelines recommend early identification of patients at risk for developing HF in order to prevent progression to clinical stages of HF [49–51]. In practice, this approach translates to the universal use of low-cost interventions such as medication (such as angiotensin-converting enzyme inhibitors or beta blockers) and lifestyle adaptation [33••]. This approach would provide benefit for various stakeholders, including patients and healthcare systems. Lifestyle changes could include improved education on HF, smoking/alcohol/illicit drug cessation, improved diet, salt restriction, weight loss, reduced fluid intake, and exercise [52]. These interventions could not only reduce premature death attributed to HF but they could have a significant impact on the patients’ quality of life by limiting the worsening of outcomes such as arrhythmia, thromboembolism, and pulmonary congestion [53]. However, despite this common-sense approach to actively manage the risk of progression in asymptomatic pre-HF patients, this approach is rarely adopted, due to limitations in identifying these patients, concerns over adverse drug reactions (in people who perceive themselves as being “healthy”), and the lack of awareness of disease progression from asymptomatic to symptomatic disease [27]. This is a great area of educational unmet need [46].

Most of the studies identified (*n* = 9; 75%) relied on echocardiography performed by trained sonographers to ascertain HF diagnosis, which is the gold standard technique for diagnosing HF according to guidelines from the American Society of Echocardiography and the European Association of Cardiovascular Imaging [54]. Acquisition of images and interpretation of echocardiograms largely depend on the skills of the professional performing the assessment and could therefore explain the variability in the estimates obtained in the various studies. Indeed, several large population-based studies have identified that the degree of variability in measurements can vary by as much as 20% due to subjectivity and inter-operative variability [55]. In five (41.7%) studies, diagnosis validated by an expert panel decreased the chance of misclassification of HF stage due to assessor variability.

To adhere to the protocol submitted to PROSPERO, this review did not include publications after March 2020. It is possible that a more recent search may observe differing results. The generalizability of the findings presented here may be limited considering that nine (75%) community-based studies were conducted in specific population subgroups defined either by age or ethnicity, and two studies had a very small sample size (*n* < 70). The impact of publication bias was minimized by the conduct of literature searches using two complementary electronic bibliographical databases and with pragmatic searches using search engines for gray literature. The assessment of the methodological quality of included studies represents a major strength of this study as it was shown to vary between observational studies.

## Conclusions

In conclusion, results from the systematic review suggest that pre-HF is a prevalent condition and most likely only a fraction of patients are diagnosed in clinical practice due to their asymptomatic condition. Higher prevalence estimates were found in elderly patients, in men, and in those with pre-existing conditions such as hypertension. Progression from pre-HF to higher symptomatic stages (HF or advanced HF) is associated with an increased mortality. Hence, early detection of patients with pre-HF and implementation of targeted interventions may reduce the burden of disease by reducing the incidence of associated complications and premature mortality.

## Supplementary Information

Below is the link to the electronic supplementary material.

Supplementary file1 (DOCX 18 KB)

Supplementary file2 (DOCX 40 KB)

Supplementary file3 (DOCX 37 KB)

## References

[1] van Riet EE, Hoes AW, Wagenaar KP, Limburg A, Landman MA, Rutten FH (2016). Epidemiology of heart failure: the prevalence of heart failure and ventricular dysfunction in older adults over time A systematic review. Eur J Heart Fail.

[2] James SL, Abate D, Abate KH, Abay SM, Abbafati C, Abbasi N (2018). Global, regional, and national incidence, prevalence, and years lived with disability for 354 diseases and injuries for 195 countries and territories, 1990–2017: a systematic analysis for the Global Burden of Disease Study 2017. The Lancet.

[3] Lippi G, Sanchis-Gomar F (2020). Global epidemiology and future trends of heart failure. AME Medical Journal.

[4.•] Yancy CW, Jessup M, Bozkurt B, Butler J, Casey DE, Drazner MH (2013). 2013 ACCF/AHA guideline for the management of heart failure: executive summary: a report of the American College of Cardiology Foundation/American Heart Association Task Force on practice guidelines. Circulation.

[5.•] Hunt SA, American College of C, American Heart Association Task Force on Practice G. ACC/AHA (2005). Guideline update for the diagnosis and management of chronic heart failure in the adult: a report of the American College of Cardiology/American Heart Association Task Force on Practice Guidelines (Writing Committee to Update the 2001 Guidelines for the Evaluation and Management of Heart Failure). J Am Coll Cardiol.

[6.•] Bozkurt B, Coats AJ, Tsutsui H, Abdelhamid M, Adamopoulos S, Albert N (2021). Universal definition and classification of heart failure: a report of the Heart Failure Society of America, Heart Failure Association of the European Society of Cardiology, Japanese Heart Failure Society and Writing Committee of the Universal Definition of Heart Failure. J Card Fail.

[7] Frigerio M, Oliva F, Turazza FM, Bonow RO (2003). Prevention and management of chronic heart failure in management of asymptomatic patients. Am J Cardiol.

[8] Ammar KA, Jacobsen SJ, Mahoney DW, Kors JA, Redfield MM, Burnett JC (2007). Prevalence and prognostic significance of heart failure stages: application of the American College of Cardiology/American Heart Association heart failure staging criteria in the community. Circulation.

[9] Lindenfeld J, Albert NM, Boehmer JP, Collins SP, Ezekowitz JA, Heart Failure Society of A (2010). HFSA 2010 comprehensive heart failure practice guideline. J Card Fail.

[10] Sara JD, Toya T, Taher R, Lerman A, Gersh B, Anavekar NS (2020). Asymptomatic left ventricle systolic dysfunction. Eur Cardiol.

[11] Echouffo-Tcheugui JB, Erqou S, Butler J, Yancy CW, Fonarow GC (2016). Assessing the risk of progression from asymptomatic left ventricular dysfunction to overt heart failure: a systematic overview and meta-analysis. JACC Heart Fail.

[12] O'Kane M, Porter D, McCann M, Julicher P, Christenson R, Oellerich M (2020). A value proposition for natriuretic peptide measurement in the assessment of patients with suspected acute heart failure. Clin Chim Acta.

[13] Roberts E, Ludman AJ, Dworzynski K, Al-Mohammad A, Cowie MR, McMurray JJ (2015). The diagnostic accuracy of the natriuretic peptides in heart failure: systematic review and diagnostic meta-analysis in the acute care setting. BMJ.

[14] Brunner-La Rocca HP, Sanders-van Wijk S (2019). Natriuretic peptides in chronic heart failure. Card Fail Rev.

[15] Januzzi JL, Chen-Tournoux AA, Christenson RH, Doros G, Hollander JE, Levy PD (2018). N-terminal pro-B-type natriuretic peptide in the emergency department: the ICON-RELOADED study. J Am Coll Cardiol.

[16] Baba M, Yoshida K, Ieda M. Clinical Applications of natriuretic peptides in heart failure and atrial fibrillation. Int J Mol Sci. 2019;20(11):2824. 10.3390/ijms20112824. 10.3390/ijms20112824 PMC6600257 31185605

[17] Luchner A, Hengstenberg C, Lowel H, Riegger GA, Schunkert H, Holmer S (2005). Effect of compensated renal dysfunction on approved heart failure markers: direct comparison of brain natriuretic peptide (BNP) and N-terminal pro-BNP. Hypertension.

[18] Muscari A, Bianchi G, Forti P, Magalotti D, Pandolfi P, Zoli M (2021). N-terminal pro B-type natriuretic peptide (NT-proBNP): a possible surrogate of biological age in the elderly people. Geroscience.

[19] Vaishnav J, Chasler JE, Lee YJ, Ndumele CE, Hu JR, Schulman SP (2020). Highest obesity category associated with largest decrease in N-terminal pro-B-type natriuretic peptide in patients hospitalized with heart failure with preserved ejection fraction. J Am Heart Assoc.

[20] Whitman IR, Vittinghoff E, DeFilippi CR, Gottdiener JS, Alonso A, Psaty BM (2019). NT-pro BNP as a mediator of the racial difference in incident atrial fibrillation and heart failure. J Am Heart Assoc.

[21] Ying W, Zhao D, Ouyang P, Subramanya V, Vaidya D, Ndumele CE (2018). Sex hormones and change in N-terminal pro-B-type natriuretic peptide levels: the multi-ethnic study of atherosclerosis. J Clin Endocrinol Metab.

[22] Yancy CW, Jessup M, Bozkurt B, Butler J, Casey DE, Colvin MM (2017). 2017 ACC/AHA/HFSA focused update of the 2013 ACCF/AHA guideline for the management of heart failure: a report of the American College of Cardiology/American Heart Association Task Force on Clinical Practice Guidelines and the Heart Failure Society of America. Circulation.

[23] National Institute for Health Care and Excellence. Chronic heart failure in adults: Diagnosis and management. 2018. Available at: https://www.nice.org.uk/guidance/ng106/resources/chronic-heart-failure-in-adults-diagnosis-and-management-pdf-66141541311685. Accessed February 2020. 30645061

[24] Ponikowski P, Voors AA, Anker SD, Bueno H, Cleland JGF, Coats AJS (2016). 2016 ESC Guidelines for the diagnosis and treatment of acute and chronic heart failure: the Task Force for the diagnosis and treatment of acute and chronic heart failure of the European Society of Cardiology (ESC)Developed with the special contribution of the Heart Failure Association (HFA) of the ESC. Eur Heart J.

[25] Gori M, Lam CS, D’Elia E, Iorio AM, Calabrese A, Canova P, et al. Integrating natriuretic peptides and diastolic dysfunction to predict adverse events in high-risk asymptomatic subjects. Eur J Prev Cardiol. 2020;28(9):937–945. 10.1177/2047487319899618. 10.1177/2047487319899618 34402871

[26] Ewald B, Ewald D, Thakkinstian A, Attia J (2008). Meta-analysis of B type natriuretic peptide and N-terminal pro B natriuretic peptide in the diagnosis of clinical heart failure and population screening for left ventricular systolic dysfunction. Intern Med J.

[27] Goldberg LR, Jessup M (2006). Stage B heart failure: management of asymptomatic left ventricular systolic dysfunction. Circulation.

[28] Moola S, Munn Z, Tufanaru C, Aromataris E, Sears K, Sfetcu R, Currie M, Qureshi R, Mattis P, Lisy K, Mu P-F. Chapter 7: Systematic reviews of etiology and risk. In: Aromataris E, Munn Z, editors. JBI Manual for Evidence Synthesis. JBI; 2020. Available from https://synthesismanual.jbi.global. Accessed January 2020.

[29] Higgins JPT, Thomas J, Chandler J, Cumpston M, Li T, Page MJ, et al. Cochrane handbook for systematic reviews of interventions; 2021. https://training.cochrane.org/handbook. Accessed January 2020.

[30] Institute of Medicine (2011). Finding what works in health care: standards for systematic reviews.

[31.••] Smeets M, Vaes B, Mamouris P, Van Den Akker M, Van Pottelbergh G, Goderis G (2019). Burden of heart failure in Flemish general practices: a registry-based study in the Intego database. BMJ Open.

[32.••] Gaborit FS, Kistorp C, Kumler T, Hassager C, Tonder N, Kober L (2019). Prevalence of early stages of heart failure in an elderly risk population: the Copenhagen Heart Failure Risk Study. Open Heart.

[33.••] Ghossein-Doha C, van Neer J, Wissink B, Breetveld NM, de Windt LJ, van Dijk AP (2017). Pre-eclampsia: an important risk factor for asymptomatic heart failure. Ultrasound Obstet Gynecol.

[34.••] Breetveld NM, Ghossein-Doha C, van Kuijk SM, van Dijk AP, van der Vlugt MJ, Heidema WM (2017). Prevalence of asymptomatic heart failure in formerly pre-eclamptic women: a cohort study. Ultrasound Obstet Gynecol.

[35.••] Mureddu GF, Agabiti N, Rizzello V, Forastiere F, Latini R, Cesaroni G (2012). Prevalence of preclinical and clinical heart failure in the elderly A population-based study in Central Italy. Eur J Heart Fail.

[36.••] Mureddu GF, Nistri S, Gori AM, Faggiano P, Fimiani B, Maggi A, et al. Awareness and appropriateness of the management of preclinical heart failure in outpatient clinics in Italy: insights from the VASTISSIMO study - evaluation of the appropriateness of the preclinical phase (stage A and stage B) of heart failure management in outpatient clinics in Italy. Monaldi Arch Chest Dis. 2019;89(1). 10.4081/monaldi.2019.1006. **This study was identified through the systematic review and the data was extracted to discuss the prevalence of pre-HF.** 10.4081/monaldi.2019.1006 30968657

[37.••] Shah AM, Claggett B, Loehr LR, Chang PP, Matsushita K, Kitzman D (2017). Heart failure stages among older adults in the community: the Atherosclerosis Risk in Communities Study. Circulation.

[38.••] Xanthakis V, Enserro DM, Larson MG, Wollert KC, Januzzi JL, Levy D (2016). Prevalence, neurohormonal correlates, and prognosis of heart failure stages in the community. JACC Heart Fail.

[39] Gupta S, Rohatgi A, Ayers CR, Patel PC, Matulevicius SA, Peshock RM (2011). Risk scores versus natriuretic peptides for identifying prevalent stage B heart failure. Am Heart J.

[40.••] Jorge AL, Rosa ML, Martins WA, Correia DM, Fernandes LC, Costa JA (2016). The prevalence of stages of heart failure in primary care: a population-based study. J Card Fail.

[41.••] Pugliese NR, Fabiani I, La Carrubba S, Carerj S, Conte L, Colonna P (2018). Prognostic value of a tissue Doppler index of systodiastolic function in patients with asymptomatic heart failure. J Cardiovasc Echogr.

[42] Miura M, Sakata Y, Nochioka K, Takada T, Tadaki S, Ushigome R (2014). Prevalence, predictors and prognosis of patients with heart failure requiring nursing care. Circ J.

[43] Benjamin EJ, Muntner P, Alonso A, Bittencourt MS, Callaway CW, Carson AP (2019). Heart Disease and Stroke Statistics-2019 update: a report from the American Heart Association. Circulation.

[44] Benjamin EJ, Blaha MJ, Chiuve SE, Cushman M, Das SR, Deo R (2017). Heart Disease and Stroke Statistics-2017 update: a report from the American Heart Association. Circulation.

[45] Yang H, Negishi K, Wang Y, Nolan M, Saito M, Marwick TH (2016). Echocardiographic screening for non-ischaemic stage B heart failure in the community. Eur J Heart Fail.

[46] Ponikowski P, Anker SD, AlHabib KF, Cowie MR, Force TL, Hu S (2014). Heart failure: preventing disease and death worldwide. ESC Heart Fail.

[47] Cleland JGF (1997). Screening for left ventricular dysfunction and chronic heart failure. Dis Manage Health Outcomes.

[48] Conrad N, Judge A, Canoy D, Tran J, Pinho-Gomes AC, Millett ERC (2019). Temporal trends and patterns in mortality after incident heart failure: a longitudinal analysis of 86000 individuals. JAMA Cardiol.

[49] Swedberg K, Cleland J, Dargie H, Drexler H, Follath F, Komajda M (2005). Guidelines for the diagnosis and treatment of chronic heart failure: executive summary (update 2005): the Task Force for the Diagnosis and Treatment of Chronic Heart Failure of the European Society of Cardiology. Eur Heart J.

[50] Hunt SA, Abraham WT, Chin MH, Feldman AM, Francis GS, Ganiats TG (2005). ACC/AHA 2005 guideline update for the diagnosis and management of chronic heart failure in the adult: a report of the American College of Cardiology/American Heart Association Task Force on Practice Guidelines (Writing Committee to Update the 2001 Guidelines for the Evaluation and Management of Heart Failure): developed in collaboration with the American College of Chest Physicians and the International Society for Heart and Lung Transplantation: endorsed by the Heart Rhythm Society. Circulation.

[51] Dickstein K, Cohen-Solal A, Filippatos G, McMurray JJ, Ponikowski P, Poole-Wilson PA (2008). ESC guidelines for the diagnosis and treatment of acute and chronic heart failure 2008: the Task Force for the diagnosis and treatment of acute and chronic heart failure 2008 of the European Society of Cardiology. Developed in collaboration with the Heart Failure Association of the ESC (HFA) and endorsed by the European Society of Intensive Care Medicine (ESICM). Eur J Heart Fail.

[52] Gibbs CR, Jackson G, Lip GY (2000). ABC of heart failure. Non-drug management BMJ.

[53] Watson RD, Gibbs CR, Lip GY (2000). ABC of heart failure. Clinical features and complications BMJ.

[54] Nagueh SF, Smiseth OA, Appleton CP, Byrd BF, Dokainish H, Edvardsen T (2016). Recommendations for the evaluation of left ventricular diastolic function by echocardiography: an update from the American Society of Echocardiography and the European Association of Cardiovascular Imaging. Eur Heart J Cardiovasc Imaging.

[55] McGowan JH, Cleland JG (2003). Reliability of reporting left ventricular systolic function by echocardiography: a systematic review of 3 methods. Am Heart J.

