# Supplementary material for: Epidemiology of Asymptomatic Pre-heart Failure: a Systematic Review
Source: Curr Heart Fail Rep. 2022 Mar 30;19(3):146–56. doi: 10.1007/s11897-022-00542-5 (PMC9177493; doi:10.1007/s11897-022-00542-5)
Supplement: Supplementary file 1 — Supplementary file1 (DOCX 18 KB) [file 11897_2022_542_MOESM1_ESM.docx]

**Epidemiology of asymptomatic pre-heart failure: A systematic review**

**Current Heart Failure Reports**

Aurore Bergamasco PharmD, MSc^1^, Anouk Déruaz Luyet PhD, MPH^2^, Nicholas D Gollop MB BCh, PhD^2^, Yola Moride PhD^1,3,4,5^, Qing Qiao MD, PhD^2^

YolaRX Consultants, Paris, France.

^2^Boehringer Ingelheim International GmbH, Ingelheim am Rhein, Germany.

^3^YolaRX Consultants, Montreal, Canada.

4Faculty of Pharmacy, Université de Montréal, Montreal, Canada.

5Rutgers, The State University of New Jersey, New Brunswick, NJ, USA.

**CORRESPONDENCE**

Aurore Bergamasco PharmD, MSc

Email: aurore.bergamasco@yolarx.com

**SUPPLEMENTARY TABLE 1** Literature search strategies (last updated on 12 March 2020)

| Search number | Parameter | Searches | Results |
| --- | --- | --- | --- |
| *MEDLINE* | | | |
| 1 | Population | Heart Failure/cl, di, dg, ep, eh, pc [Classification, Diagnosis, Diagnostic Imaging, Epidemiology, Ethnology, Prevention & Control] | 31,143 |
| 2 |  | Ventricular Dysfunction, Left/cl, co, di, dg, ep, eh [Classification, Complications, Diagnosis, Diagnostic Imaging, Epidemiology, Ethnology] | 15,972 |
| 3 |  | 1 OR 2 | 44,768 |
| 4 |  | Asymptomatic diseases/ or (untreated or undiagnosed or asymptomatic or early stage* or stage A or stage B or preclinical or subclinical or silent).ab,ti. | 633,417 |
| 5 |  | 3 AND 4 | 2,640 |
| 6 | Outcomes | Incidence/ or Prevalence/ or Risk Factors/ or Mortality/ or Disease Progression/ or Prognosis/ or Hospitalization/ or (diagnostic rate or detection rate or early detection or complication*).ab,ti. | 2,606,894 |
| 7 | Combining population and outcomes | 5 AND 6 | 1,239 |
| 8 | Limits | 7 limited to (abstracts, humans and yr="2010 -Current") | 763 |
| *Embase* | | | |
| 1 | Population | heart failure/di, ep, pc [Diagnosis, Epidemiology, Prevention] | 15,883 |
| 2 |  | heart left ventricle failure/di, ep, pc [Diagnosis, Epidemiology, Prevention] | 3,699 |
| 3 |  | 1 OR 2 | 19,190 |
| 4 |  | Asymptomatic disease/ or (untreated or undiagnosed or asymptomatic or early stage* or stage A or stage B or preclinical or subclinical or silent).ab,ti. | 904,879 |
| 5 |  | 3 AND 4 | 1,065 |
| 6 | Outcomes | Incidence/ or Prevalence/ or Risk Factor/ or Mortality/ or Prognosis/ or Hospitalization/ or (diagnostic rate or detection rate or early detection or complication*).ab,ti. | 4,134,731 |
| 7 | Combining population and outcomes | 5 AND 6 | 551 |
| 8 | Limits | 7 limited to (abstracts, humans and yr="2010 -Current") | 267 |

Abbreviation: ab,ti, abstract/title.

*truncation symbol.
