# Supplementary material for: Epidemiology of Asymptomatic Pre-heart Failure: a Systematic Review
Source: Curr Heart Fail Rep. 2022 Mar 30;19(3):146–56. doi: 10.1007/s11897-022-00542-5 (PMC9177493; doi:10.1007/s11897-022-00542-5)
Supplement: Supplementary file 2 — Supplementary file2 (DOCX 40 KB) [file 11897_2022_542_MOESM2_ESM.docx]

**Epidemiology of asymptomatic pre-heart failure: A systematic review**

**Current Heart Failure Reports**

Aurore Bergamasco PharmD, MSc^1^, Anouk Déruaz Luyet PhD, MPH^2^, Nicholas D Gollop MB BCh, PhD^2^, Yola Moride PhD^1,3,4,5^, Qing Qiao MD, PhD^2^

YolaRX Consultants, Paris, France.

^2^Boehringer Ingelheim International GmbH, Ingelheim am Rhein, Germany.

^3^YolaRX Consultants, Montreal, Canada.

4Faculty of Pharmacy, Université de Montréal, Montreal, Canada.

5Rutgers, The State University of New Jersey, New Brunswick, NJ, USA.

**CORRESPONDENCE**

Aurore Bergamasco PharmD, MSc

Email: aurore.bergamasco@yolarx.com

**SUPPLEMENTARY TABLE 2** Definition of Stage B HF as reported in publication

| **Study authors** | **Stage B HF definition** |
| --- | --- |
| Smeets M et al. [1] | Structural or functional heart disease without HF symptoms including acute myocardial infarction, heart valve disease, cardiomyopathy or tachyarrhytmia |
| Gaborit FS et al. [2] | - Stage B HF definition based on ACC/AHA guidelines - Patients with risk factors of HF, with an abnormal ECG but without past or current symptoms of HF |
| Ghossein-Doha C et al. [3] | Presence of previous myocardial infarction, LV concentric remodeling, LV hypertrophy, loss of systolic function or asymptomatic valvular disease |
| Breetveld NM et al. [4] | Presence of previous myocardial infarction, LV hypertrophy (LVMi > 95 g/m^2^), concentric remodeling (RWT > 0.42 and LVMi ≤ 95 g/m^2^), mildly impaired LVEF (>40 and <55%) or asymptomatic valvular disease |
| Pugliese N et al. [5] | Structural heart disease but without signs or symptoms of HF |
| Mureddu GF et al. [6] | Presence of a structural heart disease detected at the echocardiographic examination, or ALVD, or of a positive clinical history for cardiovascular or valvular disease in the absence of signs or symptoms of HF |
| Mureddu GF et al. [7] | - Stage B HF definition based on ACA/AHA guidelines - Previous acute myocardial infarction or chronic ischemic heart disease (stable angina or history of revascularization); stroke or transient ischemic attack; peripheral vascular disease, carotid disease, or abdominal aortic aneurysm either clinical or subclinical; chronic atrial fibrillation (permanent); LV hypertrophy at ECG (voltage-duration or Perugia strain criteria); left bundle branch block, structural LV abnormalities detected at ECG; diastolic dysfunction; any valvular heart disease at least or more than moderate; kidney chronic disease, KDOQI class ≥3 |
| Shah AM et al. [8] | Abnormal LVEF based on ARIC reference limits (<57.4% in women or <59.0% in men), regional wall motion abnormality; LV enlargement based on LVEDV indexed to BSA above ARIC reference limits (>51.9 ml/m^2^ in women or >60.2 ml/m^2^ in men); LVH based on ARIC reference limits for LV mass indexed to height^2.7^ (>41.5 g/m^2.7^ in women or >45.0 g/m^2.7^ in men); moderate or greater aortic stenosis defined as a peak transaortic velocity >3.0 m/sec; moderate or greater aortic regurgitation based on visual estimation by a staff echocardiographer; moderate or greater mitral regurgitation based on a mitral regurgitation jet area-to-left atrial ratio or >0.20; and moderate or greater mitral stenosis based on a mean antegrade transmitral gradient of at least 5 mmHg |
| Xanthakis V et al. [9] | - Stage B HF definition based on ACC/AHA guidelines - Patients with any of the following: prior myocardial infarction, valvular heart disease, or echocardiographic evidence of asymptomatic LV systolic dysfunction, hypertrophy by American Society of Echocardiography criteria, enlargement or any regional wall motion abnormality |
| Gupta S et al. [10] | Presence of LVH or LVSD or prior myocardial infarction in the absence of HF symptoms. |
| Miura M et al. [11] | Stage B HF definition based on ACC/AHA guidelines |
| Jorge AL et al. [12] | Stage B HF definition based on ACC/AHA guidelines |

Abbreviations: ACC/AHA, American College of Cardiology / American Heart Association guidelines; ALVD, Asymptomatic left ventricular dysfunction; ARIC, Atherosclerosis Risk in Communities Study; BSA, Body surface area; ECG, echocardiography; HF, Heart failure; KDOQI, Kidney Disease Outcomes Quality Initiative; LV, Left ventricular; LVEDV, LV end-diastolic volume; LVEF, Left ventricular ejection fraction; LVH, Left ventricular hypertrophy; LVMi, Left ventricular mass index; LVSD, Left ventricular systolic dysfunction; RWT, Relative wall thickness.

**References**

1. Smeets M, Vaes B, Mamouris P, Van Den Akker M, Van Pottelbergh G, Goderis G, et al. Burden of heart failure in Flemish general practices: a registry-based study in the Intego database. BMJ Open. 2019;9(1):e022972. doi: 10.1136/bmjopen-2018-022972.

2. Gaborit FS, Kistorp C, Kumler T, Hassager C, Tonder N, Kober L, et al. Prevalence of early stages of heart failure in an elderly risk population: the Copenhagen Heart Failure Risk Study. Open Heart. 2019;6(1):e000840. doi: 10.1136/openhrt-2018-000840.

3. Ghossein-Doha C, van Neer J, Wissink B, Breetveld NM, de Windt LJ, van Dijk AP, et al. Pre-eclampsia: an important risk factor for asymptomatic heart failure. Ultrasound Obstet Gynecol. 2017;49(1):143-9. doi: 10.1002/uog.17343.

4. Breetveld NM, Ghossein-Doha C, van Kuijk SM, van Dijk AP, van der Vlugt MJ, Heidema WM, et al. Prevalence of asymptomatic heart failure in formerly pre-eclamptic women: a cohort study. Ultrasound Obstet Gynecol. 2017;49(1):134-42. doi: 10.1002/uog.16014.

5. Pugliese NR, Fabiani I, La Carrubba S, Carerj S, Conte L, Colonna P, et al. Prognostic Value of a Tissue Doppler Index of Systodiastolic Function in Patients with Asymptomatic Heart Failure. J Cardiovasc Echogr. 2018;28(2):95-100. doi: 10.4103/jcecho.jcecho_59_17.

6. Mureddu GF, Agabiti N, Rizzello V, Forastiere F, Latini R, Cesaroni G, et al. Prevalence of preclinical and clinical heart failure in the elderly. A population-based study in Central Italy. Eur J Heart Fail. 2012;14(7):718-29. doi: 10.1093/eurjhf/hfs052.

7. Mureddu GF, Nistri S, Gori AM, Faggiano P, Fimiani B, Maggi A, et al. Awareness and appropriateness of the management of preclinical heart failure in outpatient clinics in Italy: Insights from the VASTISSIMO study - EValuation of the AppropriateneSs of The preclInical phase (Stage A and Stage B) of Heart FaIlure Management in Outpatient Clinics in Italy. Monaldi Arch Chest Dis. 2019;89(1). doi: 10.4081/monaldi.2019.1006.

8. Shah AM, Claggett B, Loehr LR, Chang PP, Matsushita K, Kitzman D, et al. Heart Failure Stages Among Older Adults in the Community: The Atherosclerosis Risk in Communities Study. Circulation. 2017;135(3):224-40. doi: 10.1161/CIRCULATIONAHA.116.023361.

9. Xanthakis V, Enserro DM, Larson MG, Wollert KC, Januzzi JL, Levy D, et al. Prevalence, Neurohormonal Correlates, and Prognosis of Heart Failure Stages in the Community. JACC Heart Fail. 2016;4(10):808-15. doi: 10.1016/j.jchf.2016.05.001.

10. Gupta S, Rohatgi A, Ayers CR, Patel PC, Matulevicius SA, Peshock RM, et al. Risk scores versus natriuretic peptides for identifying prevalent stage B heart failure. Am Heart J. 2011;161(5):923-30 e2. doi: 10.1016/j.ahj.2011.01.007.

11. Miura M, Sakata Y, Nochioka K, Takada T, Tadaki S, Ushigome R, et al. Prevalence, predictors and prognosis of patients with heart failure requiring nursing care. Circ J. 2014;78(9):2276-83. doi: 10.1253/circj.cj-14-0387.

12. Jorge AL, Rosa ML, Martins WA, Correia DM, Fernandes LC, Costa JA, et al. The Prevalence of Stages of Heart Failure in Primary Care: A Population-Based Study. J Card Fail. 2016;22(2):153-7. doi: 10.1016/j.cardfail.2015.10.017.
