# Supplementary material for: Epidemiology of Asymptomatic Pre-heart Failure: a Systematic Review
Source: Curr Heart Fail Rep. 2022 Mar 30;19(3):146–56. doi: 10.1007/s11897-022-00542-5 (PMC9177493; doi:10.1007/s11897-022-00542-5)
Supplement: Supplementary file 3 — Supplementary file3 (DOCX 37 KB) [file 11897_2022_542_MOESM3_ESM.docx]

**Epidemiology of asymptomatic pre-heart failure: A systematic review**

**Current Heart Failure Reports**

Aurore Bergamasco PharmD, MSc^1^, Anouk Déruaz Luyet PhD, MPH^2^, Nicholas D Gollop MB BCh, PhD^2^, Yola Moride PhD^1,3,4,5^, Qing Qiao MD, PhD^2^

YolaRX Consultants, Paris, France.

^2^Boehringer Ingelheim International GmbH, Ingelheim am Rhein, Germany.

^3^YolaRX Consultants, Montreal, Canada.

4Faculty of Pharmacy, Université de Montréal, Montreal, Canada.

5Rutgers, The State University of New Jersey, New Brunswick, NJ, USA.

**CORRESPONDENCE**

Aurore Bergamasco PharmD, MSc

Email: aurore.bergamasco@yolarx.com

**SUPPLEMENTARY TABLE 3** Quality assessment by study using JBI appraisal checklist for studies reporting prevalence data

| **Reference** | **JBI Score** | | | | | | | | | **Quality**  **score** | **Overall appraisal** |
| --- | --- | --- | --- | --- | --- | --- | --- | --- | --- | --- | --- |
|  | 1 | 2 | 3 | 4 | 5 | 6 | 7 | 8 | 9 |  |  |
| Breetveld NM et al, 2017 [1] | Yes | Yes | Yes | Yes | Yes | Yes | Yes | UC | UC | 7 | Good |
| Gaborit FS et al, 2019 [2] | Yes | Yes | Yes | Yes | Yes | Yes | Yes | UC | UC | 7 | Good |
| Ghossein-Doha C et al, 2017 [3] | Yes | Yes | UC | Yes | Yes | Yes | Yes | UC | UC | 6 | Medium |
| Gupta S et al,  2011 [4] | Yes | Yes | Yes | Yes | Yes | Yes | Yes | UC | Yes | 8 | Good |
| Jorge AL et al, 2016 [5] | Yes | Yes | UC | Yes | Yes | Yes | Yes | UC | UC | 6 | Medium |
| Mureddu GF et al, 2012 [6] | Yes | Yes | Yes | Yes | Yes | Yes | Yes | UC | Yes | 8 | Good |
| Mureddu GF et al, 2019 [7] | Yes | Yes | Yes | Yes | UC | Yes | UC | UC | UC | 5 | Medium |
| Shah AM et al, 2017 [8] | Yes | Yes | Yes | Yes | Yes | Yes | Yes | UC | Yes | 8 | Good |
| Smeets M et al, 2019 [9] | Yes | Yes | Yes | Yes | Yes | Yes | Yes | UC | Yes | 8 | Good |
| Xanthakis V et al, 2016 [10] | Yes | Yes | Yes | Yes | Yes | Yes | Yes | UC | Yes | 8 | Good |

Abbreviations: UC: Unclear.

Good overall appraisal: at least 7 of 9 items.

Medium overall appraisal: between 4 and 6 items.

**References**

1. Breetveld NM, Ghossein-Doha C, van Kuijk SM, van Dijk AP, van der Vlugt MJ, Heidema WM, et al. Prevalence of asymptomatic heart failure in formerly pre-eclamptic women: a cohort study. Ultrasound Obstet Gynecol. 2017;49(1):134-42. doi: 10.1002/uog.16014.

2. Gaborit FS, Kistorp C, Kumler T, Hassager C, Tonder N, Kober L, et al. Prevalence of early stages of heart failure in an elderly risk population: the Copenhagen Heart Failure Risk Study. Open Heart. 2019;6(1):e000840. doi: 10.1136/openhrt-2018-000840.

3. Ghossein-Doha C, van Neer J, Wissink B, Breetveld NM, de Windt LJ, van Dijk AP, et al. Pre-eclampsia: an important risk factor for asymptomatic heart failure. Ultrasound Obstet Gynecol. 2017;49(1):143-9. doi: 10.1002/uog.17343.

4. Gupta S, Rohatgi A, Ayers CR, Patel PC, Matulevicius SA, Peshock RM, et al. Risk scores versus natriuretic peptides for identifying prevalent stage B heart failure. Am Heart J. 2011;161(5):923-30 e2. doi: 10.1016/j.ahj.2011.01.007.

5. Jorge AL, Rosa ML, Martins WA, Correia DM, Fernandes LC, Costa JA, et al. The Prevalence of Stages of Heart Failure in Primary Care: A Population-Based Study. J Card Fail. 2016;22(2):153-7. doi: 10.1016/j.cardfail.2015.10.017.

6. Mureddu GF, Agabiti N, Rizzello V, Forastiere F, Latini R, Cesaroni G, et al. Prevalence of preclinical and clinical heart failure in the elderly. A population-based study in Central Italy. Eur J Heart Fail. 2012;14(7):718-29. doi: 10.1093/eurjhf/hfs052.

7. Mureddu GF, Nistri S, Gori AM, Faggiano P, Fimiani B, Maggi A, et al. Awareness and appropriateness of the management of preclinical heart failure in outpatient clinics in Italy: Insights from the VASTISSIMO study - EValuation of the AppropriateneSs of The preclInical phase (Stage A and Stage B) of Heart FaIlure Management in Outpatient Clinics in Italy. Monaldi Arch Chest Dis. 2019;89(1). doi: 10.4081/monaldi.2019.1006.

8. Shah AM, Claggett B, Loehr LR, Chang PP, Matsushita K, Kitzman D, et al. Heart Failure Stages Among Older Adults in the Community: The Atherosclerosis Risk in Communities Study. Circulation. 2017;135(3):224-40. doi: 10.1161/CIRCULATIONAHA.116.023361.

9. Smeets M, Vaes B, Mamouris P, Van Den Akker M, Van Pottelbergh G, Goderis G, et al. Burden of heart failure in Flemish general practices: a registry-based study in the Intego database. BMJ Open. 2019;9(1):e022972. doi: 10.1136/bmjopen-2018-022972.

10. Xanthakis V, Enserro DM, Larson MG, Wollert KC, Januzzi JL, Levy D, et al. Prevalence, Neurohormonal Correlates, and Prognosis of Heart Failure Stages in the Community. JACC Heart Fail. 2016;4(10):808-15. doi: 10.1016/j.jchf.2016.05.001.
